# Leveraging blood serotonin as an endophenotype to identify de novo and rare variants involved in autism

**DOI:** 10.1186/s13229-017-0130-3

**Published:** 2017-03-21

**Authors:** Rui Chen, Lea K. Davis, Stephen Guter, Qiang Wei, Suma Jacob, Melissa H. Potter, Nancy J. Cox, Edwin H. Cook, James S. Sutcliffe, Bingshan Li

**Affiliations:** 1 Molecular Physiology & Biophysics, Vanderbilt University, Nashville, TN USA; 2 Vanderbilt Genetics Institute, Vanderbilt University, Nashville, TN USA; 3 Division of Genetic Medicine, Department of Medicine, Vanderbilt University, Nashville, TN USA; 4 Department of Psychiatry, University of Illinois at Chicago, Chicago, IL USA; 5 Department of Psychiatry, University of Minnesota, Minneapolis, MN USA

**Keywords:** Autism, Autism spectrum disorder, De novo mutation, Rare variants, Compound heterozygotes, 5-HT, Serotonin, Hyperserotonemia, Endophenotype, Whole exome sequencing, Group-wise transmission/disequilibrium test

## Abstract

**Background:**

Autism spectrum disorder (ASD) is one of the most highly heritable neuropsychiatric disorders, but underlying molecular mechanisms are still unresolved due to extreme locus heterogeneity. Leveraging meaningful endophenotypes or biomarkers may be an effective strategy to reduce heterogeneity to identify novel ASD genes. Numerous lines of evidence suggest a link between hyperserotonemia, i.e., elevated serotonin (5-hydroxytryptamine or 5-HT) in whole blood, and ASD. However, the genetic determinants of blood 5-HT level and their relationship to ASD are largely unknown.

**Methods:**

In this study, pursuing the hypothesis that de novo variants (DNVs) and rare risk alleles acting in a recessive mode may play an important role in predisposition of hyperserotonemia in people with ASD, we carried out whole exome sequencing (WES) in 116 ASD parent-proband trios with most (107) probands having 5-HT measurements.

**Results:**

Combined with published ASD DNVs, we identified *USP15* as having recurrent de novo loss of function mutations and discovered evidence supporting two other known genes with recurrent DNVs (*FOXP1* and *KDM5B*). Genes harboring functional DNVs significantly overlap with functional/disease gene sets known to be involved in ASD etiology, including FMRP targets and synaptic formation and transcriptional regulation genes. We grouped the probands into High-5HT and Normal-5HT groups based on normalized serotonin levels, and used network-based gene set enrichment analysis (NGSEA) to identify novel hyperserotonemia-related ASD genes based on LoF and missense DNVs. We found enrichment in the High-5HT group for a gene network module (DAWN-1) previously implicated in ASD, and this points to the TGF-β pathway and cell junction processes. Through analysis of rare recessively acting variants (RAVs), we also found that rare compound heterozygotes (CHs) in the High-5HT group were enriched for loci in an ASD-associated gene set. Finally, we carried out rare variant group-wise transmission disequilibrium tests (gTDT) and observed significant association of rare variants in genes encoding a subset of the serotonin pathway with ASD.

**Conclusions:**

Our study identified *USP15* as a novel gene implicated in ASD based on recurrent DNVs. It also demonstrates the potential value of 5-HT as an effective endophenotype for gene discovery in ASD, and the effectiveness of this strategy needs to be further explored in studies of larger sample sizes.

**Electronic supplementary material:**

The online version of this article (doi:10.1186/s13229-017-0130-3) contains supplementary material, which is available to authorized users.

## Background

Autism spectrum disorder (ASD) is an early-onset neurodevelopmental condition with an estimated prevalence of ~1 in 68 [1]. ASD is expressed across a spectrum of severity in two core phenotypic domains: persistent deficits in social interaction and communication and restricted, repetitive behaviors and interests. ASD is highly heritable, with an estimated heritability of 64–91% based on a recent meta-analysis [2]. The genetic basis of ASD, however, is complicated by locus heterogeneity for both common allele and rare variant effects. Although common variants in aggregate contribute to a larger proportion (~50%) of liability [3], genome-wide association studies (GWAS) with thousands of subjects have not found consistent, strongly associated individual common variants [4–9]. Rare de novo variants, including both copy number (CNVs) and single nucleotide variants (SNVs), play a significant role in ASD liability [10]. To date, dozens of genes harboring de novo CNVs and SNVs meeting genome-wide significance have been identified, and corresponding functional pathways and biological processes have emerged from analysis of these variants [10]. Despite advances in identifying ASD risk loci, major hurdles remain, since rare variants account for only a minority of cases, and effect sizes for common variants necessitate GWAS sample sizes many times those currently available. Data indicate that a thousand or more genes may contribute to ASD liability [11].

In addition to larger samples, another strategy to tackle heterogeneity leverages meaningful endophenotypes or biomarkers that demonstrate heritability [12, 13]. The hypothesis that endophenotypes reflect variation in a subset of the broader set of disease risk genes leads to the notion that the subgroup of cases that share the endophenotype is more genetically homogeneous. Thus, gene discovery in such a subgroup affords greater power compared with a similarly sized group from the overall disease population and in the case of molecular traits may provide a more direct path to functional mechanisms. Biomarkers and endophenotypes in ASD have drawn interest given the potential to facilitate earlier diagnosis and better prediction of prognosis or treatment response [14].

Hyperserotonemia, or elevated serotonin (5-hydroxytryptamine or 5-HT) in whole blood, is one of the most consistent quantitative traits and biomarkers in ASD since its identification in 1961 [15–18]. In particular, these studies reported a significantly higher 5-HT blood level in about one third of ASD subjects, compared with typically developing controls. The elevated 5-HT level, or hyperserotonemia, is observed in ASD but not in subjects with unspecified intellectual disability [19]. Whole blood 5-HT levels show intermediate elevation in first-degree relatives of hyperserotonemic probands [20–23]. While hyperserotonemia in ASD shows evidence for heritability, whole blood 5-HT also exhibited high narrow and broad heritability (0.51 and 1.0, respectively) in a Hutterite population sample [24]. Although the mechanism underlying the elevation of serotonin levels in ASD remains unclear, several lines of investigation point to a role for serotonin in ASD etiology [25–29]. In blood, greater than 99% of the serotonin is stored in platelets, which is taken up from the enterohepatic circulation by the serotonin transporter (SERT), encoded by *SLC6A4*, after synthesis in enterochromaffin cells of the gut. Linkage studies in ASD have implicated the 17q11.2 region harboring *SLC6A4* [30–32]. Hypothesizing rare variants in the absence of significant allelic association at *SLC6A4* led to the discovery of multiple functional coding variants [30], and in particular, the association of *SERT* Ala56 was supported in mice by the evidence that mice carrying the variant displayed alterations in social function, communication, and repetitive behavior and elevated whole blood 5-HT [33]. These findings collectively support hyperserotonemia as a powerful endophenotype for dissecting the genetic etiology of ASD.

In this study, we carried out whole exome sequencing (WES) in a collection of ASD parent-proband trios with 5-HT measurements collected through an Autism Center of Excellence (ACE) study to search for genetic variants implicated in ASD using serotonin as an endophenotype. Given that elevated serotonin was observed in ASD probands compared with their parents, we hypothesize that de novo variants (DNVs) and recessively acting variants (RAVs) play a key role in predisposition of hyperserotonemia and ASD. DNVs observed in probands, but not parents, which disrupt genes involved in 5-HT and ASD, should affect 5-HT levels only in probands; similarly, rare risk alleles acting in a recessive manner (i.e., RAVs) that are transmitted from parents to probands may lead to elevated 5-HT levels in those probands. Both DNVs and RAVs have been implicated in ASD [34]; however, the allelic architecture of hyperserotonemia in autism is unknown. We thus aimed to utilize this unique endophenotype in ACE trios to identify genes involved in both traits; an approach that we hypothesize effectively reduces genetic heterogeneity. Moreover, we predict that positive findings will shed light on what serotonin-related functional pathways are involved in ASD. Corresponding genes and proteins may offer insights into dysregulated CNS development and point to therapeutic strategies for ASD symptoms.

## Methods

### Whole exome sequencing and data processing

Exome capture and sequencing were conducted at the Center for Inherited Disease Research (CIDR) and HudsonAlpha (11 trios). Genomic DNA was extracted from either cell line or blood samples collected from all participating family members in ACE. At HudsonAlpha, exons were captured using NimbleGen Seq Cap EZ SR v2 (Roche, Switzerland) and sequenced on an IlluminaHiSeq2000 (Illumina, USA). At CIDR, exons were captured using Agilent SureSelect XT HumanAllExon V5 + UTRs_71MbKit_S04380219 (Agilent Technologies, USA). Sequencing platform and chemistry was Illumina HiSeq2500 (Illumina, USA), TruSeq Rapid PE Cluster Kit-HS (Illumina, USA), and TruSeq Rapid SBS-HS (Illumina, USA) with 100 bp paired-end runs at ~60×. Sequencing reads were aligned to the reference human genome GRCh37 using BWA [35] with default settings. Next, the resulting BAM files were further processed and used for variant calling following the best practice procedures of GATK [36, 37]. We retained biallelic SNVs with PASS VQSR tag and average depth >10. We masked genotypes of trios with minimal depth <6 as missing. We annotated all variants using ANNOVAR [38] and obtained deleteriousness predictions from nine bioinformatics tools provided in ANNOVAR. Each variant was assigned a deleterious score (DScore) between 0 and 9, denoting the number of damaging predictions from among these algorithms. We used TrioDenovo to call DNVs on both autosomes and X chromosome and obtained reliable candidates for validation after filtering [39]. We focused on two types of functional variants: loss of function (LoF; splice site, nonsense, and frameshift) and missense (Mis) DNVs. We experimentally validated all functional DNVs by Sanger sequencing and obtained 100 validated functional DNVs, including 16 LoF and 84 Mis DNVs across 99 genes (Additional file 1: Table S1).

In RAV analysis, we obtained 2073 LoF and 39,095 Mis-D4 SNVs after filtering. To get reliable RAVs, we used the phase-by-transmission algorithm to construct haplotypes in trios to achieve robust phasing for rare variants [40].

### Normalization of whole blood serotonin

In order to compare whole blood serotonin (WB5HT) values across age and population groups, z-scores were generated to adjust for pubertal status and race/ethnicity. Pubertal status was determined based on their Tanner stage [41, 42] or chronological age. Subjects were classified as pre-pubertal if their Tanner stage was either I or II and post-pubertal if their Tanner stage was greater than or equal to III. In the case of missing Tanner data, chronological age was used to create the puberty variable. Subjects with a chronological age less than 144 months were classified as pre-pubertal, whereas subjects whose chronological age were greater than or equal to 144 months were classified as post-pubertal.

Race and ethnicity information was obtained during an initial screening by parent report. A subject’s reported race and ethnicity was classified based on self-report and confirmed by principal components analysis (PCA). Utilizing the respective ancestry group, means and standard deviations (SD) for WB5HT of the pre-pubertal, “normal control” children generated previously, WB5HT z-scores were calculated for our pre-pubertal subjects. For post-pubertal subjects, WB5HT z-scores were generated from the WB5HT means and SDs of the adult subjects in [43] (see Additional file 1: Table S2). For subjects with more than one reported race/ethnicity, the average of the respective means and SDs were used to calculate their z-score. Individual subjects taking medications that could potentially influence serotonin as well as other psychotropic medications were excluded from WB5HT analyses.

After normalization, we separated probands into two groups based on the distribution of WB5HT-z in parents and probands (Additional file 2: Figure S1). Specifically, we classified probands with WB5HT-z >1.75 as the High-5HT group and probands with WB5HT-z <1 as the Normal-5HT group, with 30 and 67 probands in the High-5HT and Normal-5HT groups, respectively. For analysis of WB5HT-z subgroups, we did not include the 10 probands (and corresponding genes) who had values between 1 and 1.75 to increase separation between comparison groups, given the absence of an obvious cut-point for defining hyperserotonemia.

### RAV burden in High-5HT group vs. Normal-5HT groups

For RAV analysis, we carried out PCA on our data combined with the 1000 Genomes Project data and selected probands of Western European descent (25 High-5HT and 38 Normal-5HT) based on the first two PCs to avoid confounds caused by population stratification (Additional file 3: Figure S2). We called two types of RAVs from our data: compound heterozygotes (CHs) and homozygotes. A total of 179 RAVs (137 CH and 42 homozygote genotypes—and corresponding 155 genes—were present in 78 WE probands. Of these RAV genes, 127 are expressed (RPKM >1) in early fetal brain using data from Brainspan (Brainspan: http://www.brainspan.org/).

For each gene detected, we carried out the Fisher exact test of RAVs in the High-5HT vs. the Normal-5HT groups. Given the low power to detect significant associations of individual genes, we selected genes with an odds ratio >1 as candidates for the risk RAV genes in the High-5HT group and used genes with OR <1 as controls.

### Gene set enrichment analysis

We identified several functional/disease gene sets in ASD for gene set enrichment analysis (GSEA): (1) two lists of RBFOX1 (RNA-binding protein, fox-1 homolog 1)-regulating RNA targets: RBFOX1-1 [44]; RBFOX1-2 [45]; (2) two lists of fragile X mental retardation protein (FMRP) mRNA targets: FMRP-1 [46]; FMRP-2 [47]; (3) evolutionarily constrained genes (ECGs) [48]; (4) synapse-related gene lists: postsynapse-related genes from proteomic profiling of human neocortical biopsies (Hpsd), protein complexes of the postsynaptic density (PSD; PSD-95, ARC, mGluR5, NMDAR), and genes related to presynaptic proteins, presynaptic active zone, and synaptic vesicles [49, 50]; (5) chromatin remodeling factors (CRFs) [51]; (6) histone modification enzymes (HMEs) [52]; (7) differentially expressed genes from cortical samples of autism (DEs) [44]. All genes including candidates and known gene sets were mapped to current human gene symbols (HGNC) to assure the consistency of nomenclature.

It is well known that, generally, brain-expressed genes tend to be longer, and if a statistic is influenced by gene length, then gene length is a potentially confounding factor for GSEA (Additional file 4: Figure S3). For both DNVs and RAVs, to control for the confounding nature of gene length, we used a sampling scheme to obtain empirical *p* values. Specifically, we constructed a null distribution of the number of overlapping genes by randomly sampling genes matching the length of the candidate genes and then calculated empirical *p* values by comparing the observed overlapping gene number to the null distribution. To select a gene with matching length, we sorted genes by their coding sequence (CDS) lengths and randomly picked a gene from the neighbors of target gene. The interval of neighbors is a parameter that may affect the results, e.g., an interval of 30 means that we chose a gene from a subset of genes consisting of 30 shorter and 30 longer neighbors of the target gene. We therefore tested simulated configurations with 30, 100, or 1000 neighbors and obtained similar results across all simulated sets. We present the results using an interval of 100.

### Network-based gene set enrichment analysis

Our approach to the network-based gene set enrichment analysis (NGSEA) is based on the guilt-by-association principle, in that genes underlying risk for a given disease tend to function together in one or more functional, biological networks. This assumption underlies many approaches that have been successfully applied to search for disease genes in numerous disorders, including ASD [53–55]. Given the extensive heterogeneity of ASD, it is expected that several different networks underlying its etiology exist. To test the hypothesis that a candidate (ACE) gene set is enriched for associated genes, we reframed this hypothesis as being that novel gene candidates identified from our ACE sample are closer to established ASD genes in the network than expected by chance. Accordingly, we devised a network-based approach to measure the distance between two gene sets, similar to EnrichNet [56], and used a sampling scheme to assess the significance. The framework assigns genes in a known ASD gene set as seeds in a functional protein-protein interaction network and then runs label propagation in the network using a random walk with restart [57, 58]. Briefly, let *A* be an adjacent matrix representing the gene-gene network, with 1 in the matrix indicating that the corresponding two genes interact and 0 otherwise, *m* be the number of genes in the network, *F*_0_ be a vector of length *m* with the *i*th element being 1 if the *i*th gene is a seed gene and 0 otherwise. Label propagation is executed as *F*_t+1_ = (1 − *α*) *× A × F*_t_ + *α × F*_0_ until convergence, with *α* being the restart rate. Following propagation, each value in *F* represents the influence (i.e., the amount of flow) the corresponding gene receives from the seeds; we termed this influence the propagation-based distance in network (PDN) to measure the distance between a candidate gene and a known ASD gene set (i.e., the seeds). Next, we summed the PDN of all novel candidate genes to a known ASD gene set and used the sum as a measurement of the distance between the candidate set and the known gene set. Finally, we generated pseudo-candidate gene sets by randomly sampling genes from the human genome, controlling for gene length as in the GSEA, and then calculated the sum of the PDN between pseudo sets to the known ASD gene set to build the null distribution; this in turn permits the generation of empirical *p* values. We used the PINA [59] network in this analysis with a restart probability of 0.3.

### Rare variant group-wise transmission/disequilibrium test

We collected a set of serotonin-related genes, representing both pre- and postsynaptic functions, including 5-HT metabolism, transport, and signaling (Additional file 1: Table S3). We assigned genes in 5-HT receptor family as a group (Receptor group) and genes related to 5-HT metabolism, transport, and other largely presynaptic functions as the other group (Non-Receptor group) due to their distinct mechanisms. We carried out group-wise transmission/disequilibrium test (gTDT) on each of the two groups to test whether rare variants in these genes are associated with ASD. We kept all LoF and Mis-D4 (Dscore ≥4) variants with MAF <0.01 located on autosomes; in this analysis we used a more stringent criterion of Mis-D4 than Mis-D2 used in DNVs to filter missense variants here to potentially enrich for causal variants.

## Results

### Brief description of samples and sequence data processing

**Fig. 1:**
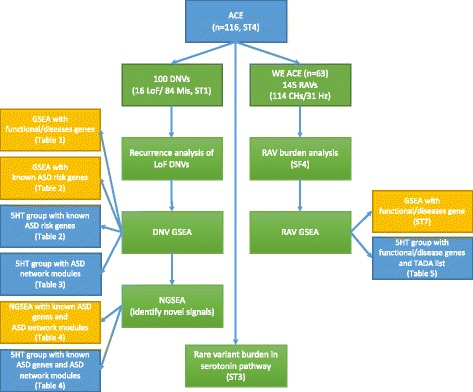
Overview of the analysis. This study shows the analysis of DNVs and RAVs in 116 families with serotonin measurement. The major steps are shown in the middle (*green*). The analysis steps identifying ASD-related signals used the whole DNV/RAV list (*yellow*). The steps identifying 5HT-related signals in ASD used DNVs/RAVs in High-5HT vs. Normal-5HT groups (*blue*). ACE, Autism Center of Excellence; DNV, de novo variant; RAV, recessive acting variant; LoF, loss of function; GSEA, Gene Set Enrichment Analysis; NGSEA, Network-based Gene Set Enrichment Analysis; 5HT, 5-hydroxytryptamine; TADA, a list from a recent study of both transmitted and de novo variants in ASD [11]; CH, compound heterozygote; Hz, homozygote

We sequenced the exomes of 133 trios as a part of the University of Illinois at Chicago ACE project. To avoid confounding our analysis with data from earlier studies, 17 trios that were included in previously published data [60] were excluded, and the remaining 116 trios were used in subsequent analyses. Detailed information on subject ascertainment and clinical assessment has been described elsewhere [61]. For this analysis, one subject with Fragile X syndrome was excluded but three subjects with clinically significant CNVs (*A2BP1*/*RBFOX1* deletion [62] (1.0 < WB5HT-z < 1.75), maternal interstitial duplication 15q11-q13 (Normal-5HT group), and *NRXN3* deletion (Normal-5HT group)) were included. Demographic and phenotypic data are summarized in Additional file 1: Table S4. As expected, males were overrepresented with a ratio of 5.44:1 (male:female), which is comparable to the general ASD population [63]. Of the 116 analyzed trios, 107 probands, 72 fathers and 63 mothers had 5-HT measurements. An overview of analyses conducted in this study is presented in Fig. 1.

### Analysis of de novo variants

#### Recurrence of LoF DNVs

We first focused on LoF DNVs as it is well established that rare LoF DNVs can exert large effect sizes on risk of ASD and are more readily detected compared with other types of variants [11, 64]. Considering the stronger purifying selection conferred by LoF mutations, recurrence of rare LoF DNVs in a specific gene is a strong indicator of disease association, and recurrence of two or more LoF DNVs in a gene typically reaches genome-wide significance for a sample size up to 3000 families [65]. We did not observe genes with recurrent LoF DNVs in our data given the limited sample size. We then compared genes implicated by our DNVs to those from other recent studies, such as the 192 LoF DNVs from the Autism Sequencing Consortium (ASC) [11] and 391 LoF DNVs from the Simons Simplex Collection (SSC) [64]. When combined with previous datasets, our study identified a second LoF DNV in ubiquitin-specific peptidase 15 (*USP15*) making this the first reported recurrence of LoF DNV in this gene, implicating it as a novel ASD candidate. A frameshift DNV was also identified in *FOXP1* (forkhead box P1) and a stop-gain mutation in *KDM5B* (lysine demethylase 5B), adding further support for these two loci already identified as having recurrent, independent DNVs (*N* ≥ 2) within SSC or ASC data. Thus, our studies provide strong support for three loci as high confidence ASD (hcASD) genes based on LoF DNVs.

#### Enrichment analysis of DNVs in functional gene sets

We restricted analysis to the genes that are expressed at early stages of brain development (Brainspan, http://www.brainspan.org/) using a criterion of RPMK >1 (﻿16 LoFs and 70 Mis). We first focused on LoF DNVs and the results are shown in Table 1. Note that for the analyses carried out in this study, we report the nominal *p* values, not corrected for multiple comparisons. We found the most significant enrichment of LoF DNV genes in the HME gene set (*p* = 0.009), of which *KDM5B* is a notable example. LoF DNV loci did not show significant enrichment in the two FMRP target gene sets (*p* = 0.415/0.157). FMRP functions as a translational repressor in neurons, and loss of FMRP function causes fragile X syndrome [66–68]. RBFOX1 (previously known as Ataxin-2-binding protein 1, A2BP1) corresponds to the only known splicing factor implicated in ASD, and hemizygous loss of *RBFOX1* is associated with ASD and multiple epilepsy syndromes [69]. However, we saw no significant enrichment among its targets (*p =* 1 and 0.522). We also did not observe significance enrichment of LoF DNVs in genes under evolutionary constraint (ECGs; *p =* 0.132) and synaptic protein gene sets.

**Table 1:** GSEA of DNVs in functional/disease clusters implicated in ASD (italics: *p* < 0.05)

	Func (86)^a^	LoFs (16)	Mis (70)
RBFOX1-1 (186)	2 (0.342)	0 (1)	2 (0.318)
RBFOX1-2 (547)	5 (0.469)	1 (0.522)	4 (0.542)
FMRP-1 (936)	*15 (0.045)*	2 (0.415)	*13 (0.045)*
FMRP-2 (831)	13 (0.136)	3 (0.157)	10 (0.256)
ECGs (928)	10 (0.341)	3 (0.132)	7 (0.598)
Hpsd (1429)	*15 (0.021)*	3 (0.181)	*12 (0.027)*
PSD-95 (107)	1 (0.392)	0 (1)	1 (0.348)
ARC (25)	0 (1)	0 (1)	0 (1)
mGluR5 (37)	0 (1)	0 (1)	0 (1)
NMDAR (59)	0 (1)	0 (1)	0 (1)
Presynaptic active zone (204)	1 (0.498)	0 (1)	1 (0.457)
Presynaptic (330)	3 (0.252)	1 (0.239)	2 (0.421)
Vesicles (104)	1 (0.367)	0 (1)	1 (0.281)
CRFs (55)	1 (0.358)	0 (1)	1 (0.361)
HMEs (146)	*4 (0.023)*	*2 (0.009)*	2 (0.301)
DEs (411)	2 (0.461)	1 (0.268)	1 (0.707)

^a^The number in the header and row names denotes the size of the list. The number of overlapped genes between candidate DNVs and functional/diseases gene sets and *p* value (in brackets) are listed in main cells

In testing for enrichment of missense DNV-containing genes with the same functional gene sets, we observed enrichment in FMRP targets and Hpsd (Table 1). When combining missense and LoF DNVs together, significance for FMRP, Hpsd, and HME gene was observed (Table 1). We then combined the LoF DNVs with the SSC and ASC studies to get a comprehensive picture of enrichment in functional/disease gene sets. Consistent results are shown in Additional file 1: Table S5, i.e. RBFOX1-2, FMRP targets, ECGs, Presynaptic genes, and HMEs show strong levels of significance.

#### Gene set enrichment analysis of DNVs in known ASD risk gene sets

Next we investigated to what degree our DNVs identify known ASD risk genes. We collected two ASD gene sets: (1) candidates identified from allelic association studies, rare single gene variants (CNVs and SNVs), and genes linked to syndromic autism, all of which are cataloged in AutDB updated on June, 2015 [70]; (2) genes with recurrent DNVs combining data from ASC and SSC (Recur) [11, 64]—we consider the latter to represent a carefully chosen set of hcASD risk genes. Indeed, we found that genes with LoF DNVs are significantly enriched amongst Recur (*p =* 3.817 × 10^−4^) genes (Table 2). The results confirm that LoF DNVs in the ACE trio sample correspond to genes with extant evidence for involvement in ASD risk. In contrast, Mis-D2 DNVs only showed a nonsignificant trend in Recur (*p =* 0.096) and no evidence for enrichment among AutDB genes (*p =* 0.161) (Table 2).

**Table 2:** GSEA of DNVs with known ASD risk gene sets (italics: *p* < 0.05)

	AutDB (781)	Recur (37)
LoFs (16)	2 (0.124)	*2 (3.817e−04)*
Mis-D2 (56)	4 (0.161)	1 (0.096)
High-5HT_LoFs (5)	0 (1.000)	0 (1.000)
High-5HT_Mis-D2 (10)	0 (1.000)	0 (1.000)
Normal-5HT_LoFs (8)	*2 (0.036)*	*2 (8.989e−05)*
Normal-5HT_Mis-D2 (38)	3 (0.179)	1 (0.067)

We asked how the signals shown above relate to the distribution of whole blood serotonin by comparing GSEA results of DNV genes in the High-5HT and Normal-5HT groups. Since serotonin level is influenced by several factors like ancestry, age, and pubertal status, we first calculated a normalized z-score for each of the probands, adjusting for ethnicity and age, and assigned to High-5HT or Normal-5HT groups based on a logical split in the distribution (“Methods”). The corresponding gene lists are provided in Additional file 1: Table S6. While the LoF DNVs in the normal-5HT group are significantly enriched in both ASD-associated genes (AutDB: *p =* 0.036, Recur: *p =* 8.989 × 10^−5^), LoF DNV genes in the High group showed no such evidence of enrichment (Table 2). Contrasting the Normal-5HT group, we observed no significant enrichment of Mis-D2 DNV genes in the High-5HT group (Table 2).

To further explore our data and map observed DNVs onto biological networks, we tested two gene network constructs previously employed to identify gene networks associated with ASD based on enrichment of rare, damaging variants: three modules (denoted as MAGI-1, 2, 3; for *Merging Affected Genes into Integrated networks*) reported in [55] and four modules (denoted as DAWN-1, 2, 3, 4, for *Detecting Association with Networks*) reported in [11]. Testing genes with de novo LoF or Mis-D2 variants from High-5HT (*N* = 5, 10) or Normal-5HT (*N* = 8, 38) groups against the three modules in the MAGI classification, showed no significant enrichment (Table 3). However, using the DAWN modules against DNV loci from the Normal-5HT group, we found significant overlap of LoF genes in DAWN-4 (*p =* 9.216 × 10^−6^), Mis-D2 genes in DAWN-1 (*p =* 0.035) and DAWN-3 (*p =* 5.280 × 10^−3^) (Table 3). This suggests that genes harboring functional DNVs from the (major) normal 5-HT part of the ACE cohort are enriched for ASD genes falling into these particular modules. In contrast, no evidence for enrichment was detected for the High-5HT group.

**Table 3:** GSEA of DNVs in High-5HT and Normal-5HT groups with network modules implicated in ASD (italics: *p* < 0.05)

	MAGI1 (80)	MAGI2 (24)	MAGI3 (91)	DAWN1 (19)	DAWN2 (20)	DAWN3 (58)	DAWN4 (113)
LoFs (16)	0 (1.000)	0 (1.000)	0 (1.000)	0 (1.000)	0 (1.000)	0 (1.000)	*3 (8.921e−05)*
Mis-Ds2 (56)	1 (0.199)	0 (1.000)	0 (1.000)	1 (0.051)	0 (1.000)	*2 (0.011)*	0 (1.000)
High-5HT_LoFs (5)	0 (1.000)	0 (1.000)	0 (1.000)	0 (1.000)	0 (1.000)	0 (1.000)	0 (1.000)
High-5HT_Mis-D2(10)	0 (1.000)	0 (1.000)	0 (1.000)	0 (1.000)	0 (1.000)	0 (1.000)	0 (1.000)
Normal-5HT_LoFs (8)	0 (1.000)	0 (1.000)	0 (1.000)	0 (1.000)	0 (1.000)	0 (1.000)	*3 (9.216e−06)*
Normal-5HT_Mis-D2 (38)	1 (0.140)	0 (1.000)	0 (1.000)	*1 (0.035)*	0 (1.000)	*2 (5.280e−03)*	0 (1.000)

#### Identification of novel ASD genes through network-based analysis of DNVs

We were particularly interested in assessing to what degree the novel DNV genes identified in our study ostensibly contribute to ASD risk, which cannot be assessed by GSEA, and thus applied NGSEA to further this goal. We note that NGSEA in this section used the same functional/disease gene sets and network modules, with the exception that the current analyses specifically test for novel signals in network space, while traditional enrichment analysis does not. No enrichment, however, was detected considering either Recur or AutDB sets, or the seven total MAGI/DAWN modules, for both LoF and Mis-D2 DNV genes (Table 4).

**Table 4:** NGSEA to identify novel signals in different groups of DNVs (italics: *p* < 0.05)

	AutDB	Recur	MAGI1	MAGI2	MAGI3	DAWN1	DAWN2	DAWN3	DAWN4
LoFs	0.838	0.07	0.225	0.326	0.224	0.187	0.52	0.502	0.748
Mis-D2	0.762	0.135	*0.026*	0.347	0.371	0.083	0.274	0.353	0.055
High-5HT_Func	0.734	0.467	0.178	0.759	0.83	*0.027*	0.335	0.376	0.244
High-5HT_LoFs	0.925	0.234	0.746	0.713	0.637	*0.039*	0.653	0.82	0.755
Normal-5HT_LoFs	0.481	0.046	0.139	0.147	0.104	0.33	0.801	0.233	0.506
High-5HT_Mis-D2	0.552	0.512	0.098	0.637	0.748	*0.04*	0.187	0.235	0.137
Normal-5HT_Mis-D2	0.612	0.1	*0.031*	0.249	0.25	0.174	0.207	0.329	0.035

**Fig. 2:**
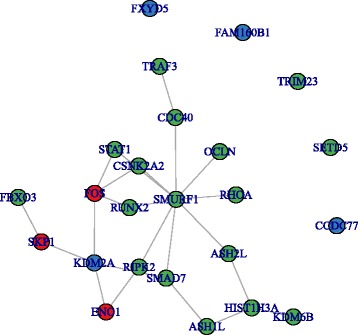
Network plot between High-5HT LoF DNV genes and the genes in DAWN-1 module. *Blue*: High-5HT LoF DNV genes; *green*: genes in DAWN-1 module; *red*: neighbor genes that connect LoF DNV genes and genes in DAWN-1. The network shown is PINA

Next we aim to inspect whether NGSEA can discover novel signals with the aid of whole blood 5-HT as an endophenotype. Results show that neither group of LoF genes were significantly closer to any of the MAGI modules than expected by chance, with only the exception that Mis-D2 of Normal-5HT group is significantly closer to MAGI-1 (Table 4). We did, however, observe significance for DAWN-1 LoF genes in the High-5HT group (*p =* 0.039, Fig. 2); Normal-5HT LoF loci were not associated with any DAWN modules (Table 4). Relating results from GSEA and NGSEA analyses, data indicate that High-5HT and Normal-5HT LoF genes identify different modules, i.e., High-5HT LoF genes identify DAWN-1 using NGSEA (Table 4), and the Normal-5HT LoF genes detect DAWN-4 using GSEA (Table 3). The complete list of the LoF DNV genes in the High-5HT group is presented in Additional file 1: Table S6 and their enrichment findings for different gene modules are shown in Table 4.

**Fig. 3:**
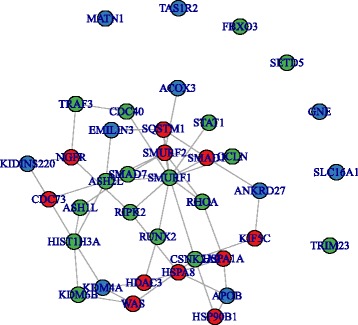
Network plot between High-5HT Mis-D2 DNV genes and the genes in DAWN-1 module. *Blue*: High-5HT Mis-D2 DNV genes; *green*: genes in DAWN-1; *red*: neighbor genes that connect Mis-D2 and DAWN-1. The network used is PINA

Like with LoF mutations, NGSEA of Mis-D2 DNV genes from the High-5HT group also revealed enrichment with DAWN-1 (*p =* 0.04, Fig. 3). However, the Normal-5HT Mis-D2 genes still showed no enrichment to DAWN modules (Table 4). In contrast to the consistent pattern of NGSEA enrichment of High-5HT DNV genes among DAWN-1, genes identified by Normal-5HT DNVs, which showed no enrichment using NGSEA, were enriched in DAWN-1, DAWN-3, and DAWN-4 modules in GSEA.

### Analysis of rare recessively acting variants

In parallel with DNVs, we hypothesize that the elevated 5-HT in probands compared to their parents is partially accounted for by rare recessively acting variants (RAVs), consisting of compound heterozygotes (CHs) and homozygotes. We focused on functional variants, comprising all LoF and Mis-D2 variants on autosomes with minor allele frequency (MAF) <0.01; we excluded X chromosomes in the RAV analysis due to its inapplicability in males. We used the phase-by-transmission algorithm to construct haplotypes in trios to achieve robust phasing for rare variants [40], and in total, we obtained 137 CHs and 42 homozygotes in probands, distributed in 155 genes. We combined both the CHs and homozygotes for the analysis of RAVs.

#### RAV burden associated with ASD

We first examined whether probands carry a higher burden of RAVs compared to their parents. As the number of RAVs in a given sample and across samples is influenced by sequencing data in complex ways, we normalized functional RAV burden using synonymous RAV burden as a baseline, assuming that synonymous variants do not (by and large) confer ASD risk. Results show that the burden of functional RAVs in probands is not significantly different from that in parents (Additional file 5: Figure S4). We further examined whether genes with RAVs in probands are enriched in functional/disease gene sets in ASD. After correcting for gene length, RAV gene sets showed no evidence to support enrichment amongst the various functional/disease gene sets (Additional file 1: Table S7), likely due to some combination of small sample size and predicted weaker effects of RAVs (relative to DNVs) in conferring ASD risk.

#### RAVs associated with hyperserotonemia

We applied GSEA to selected functional RAVs that, we envisioned, are more likely to be enriched in the High-5HT group (“Methods”) on the same functional/disease gene sets, correcting for the gene length bias. We observed no significant enrichment among any gene sets, except a trend (*p =* 0.051) towards significance in FMRP targets (Table 5). We then used a gene list corresponding to loci from a recent study of both transmitted and de novo variants in ASD [11]. Termed TADA-1, this analysis from the Autism Sequencing Consortium identified a set of 33 hcASD risk loci. Using this gene set, we observed significant enrichment for RAV-containing genes from this ACE sample (*p =* 0.03) (Table 5). The significance level is not as striking as that observed for DNVs (Table 1), likely owing to the smaller sample size of the WE subset of ACE and the weaker effect of RAVs compared to those from DNVs. Two genes, electron transfer flavoprotein beta subunit (*ETFB*) and reelin (*RELN*), from the TADA-1 comparison were found amongst the RAV gene set, and this remained significant after correction for gene length. We also carried out GSEA with a list termed TADA-2, an extended list of 65 hcASD risk loci (63 are expressed at early stages of brain development) which integrated small de novo deletions with data of TADA-1 [10]. We did not observe significance in this gene set (*p =* 0.34), with a possible reason that the gene RELN, which is one of the two overlapping genes in 33 TADA-1 hcASD risk genes, is not included in the 65 TADA-2 genes. Of note, RELN has been implicated in ASD based on numerous studies [71]. As a control, we also performed GSEA on the RAVs that are enriched in Normal-5HT group (“Methods”), and there is no enrichment in all functional/disease gene sets (Additional file 1: Table S7). We further carried out network-based enrichment analysis of the candidate RAV genes, using the same two sets of network modules and did not observe the enrichment patterns observed for DNVs.

**Table 5:** GSEA of RAVs in the High-5HT group before and after correcting for gene length (italics: *p* < 0.05)

	Before correction	After correction
RBFOX1-1 (186)	1 (0.413)	0.5
RBFOX1-2 (547)	3 (0.204)	0.57
FMRP-1 (936)	5 (0.126)	0.68
FMRP-2 (831)	10 (*9.108e−05*)	0.051
ECGs (928)	3 (0.497)	0.95
Hpsd (1429)	5 (0.385)	0.46
PSD-95 (107)	0 (1.000)	1
ARC (25)	0 (1.000)	1
mGluR5 (37)	1 (0.100)	0.18
NMDAR (59)	0 (1.000)	1
Presynaptic active zone (204)	0 (1.000)	1
Presynaptic (330)	2 (0.242)	0.24
Vesicles (104)	0 (1.000)	1
CRFs (55)	1 (0.145)	0.26
HMEs (146)	0 (1.000)	1
DEs (411)	1 (0.695)	0.57
TADA-1 (33)	2 (3.958e*−*03)	0.03
TADA-2 (63)	1 (0.165)	0.34

#### RAV burden in serotonin pathway genes

We then tested whether rare variants in serotonin pathway genes were associated with ASD by carrying out the gTDT. Because receptors represent one major category of proteins, we elected to select this functional axis as the basis for subdividing 5-HT-related genes for tests of transmission. Thus, we tested 5HT-receptor genes as one group and the remaining genes as the Non-Receptor group (“Methods”). We observed a significant over-transmission of rare functional alleles in the Non-Receptor group of genes (Transmitted: Non-Transmission = 26:13, *p =* 0.042), *which largely encode presynaptic proteins* (e.g., *SLC6A4*, *ITGB3*). In contrast to functional variants, synonymous variants exhibit approximately equal transmissions, consistent with the null expectation (62:60, *p =* 0.865). Functional variants in the Receptor group showed significant under-transmission (1:9, *p =* 0.021), although the total allele count is small. In contrast, there is no evidence for transmission distortion in the Receptor group for synonymous variants (28:36, *p =* 0.346). We also carried out gTDT on the gene set combining both Receptor and Non-Receptor groups and did not observe significance (*p =* 0.484).

## Discussion

ASD is a genetically heterogeneous disorder with estimates of 1000 or more genes involved in disease etiology. This heterogeneity poses great challenges to identify individually significant risk loci. This challenge is particularly pronounced for DNVs, as mutation rates are extremely low to observe independent de novo mutations in the same gene in a given cohort. LoF DNVs in ASD probands, although rare, are likely to have large effects when predisposing ASD risk, and therefore more likely to identify risk genes. Accordingly, when recurrence of LoF DNM in the same genes is seen in a cohort of probands, it is a strong indicator of that gene’s contribution to ASD risk. In this study, we have a very limited sample size compared to other consortium-level datasets, and unsurprisingly did not observe recurrent/independent DNVs in a gene within our data. Instead, we combined our DNVs with those from previous studies for recurrence analysis. We identified one new recurrent gene, *USP15*, as a novel ASD candidate gene, and provided further supporting evidence for two other known recurrent DNV genes (*FOXP1* and *KDM5B*). *FOXP1* has been linked to several cognitive disorders, and its deletion causes autism-like behaviors in mice [72]. *KDM5B* harbored LoF DNVs in each of two other study cohorts (two in SSC, one in ASC), and probands with *KDM5B* LoF DNVs were shown to have lower non-verbal IQ [64]. We note that de novo LoF DNVs in *KDM5B* were also observed in two unaffected (unrelated) siblings in the SSC, suggesting incomplete penetrance. USP15 acts as deubiquitinating enzyme on transforming growth factor-beta (TGF-β) and bone morphogenetic protein (BMP) stimulated R-SMADs (receptor-regulated intracellular proteins that transduce extracellular signals). We note that both TGF-β and BMP signaling are involved in differentiation of serotonergic neurons [73], but the role of *USP15* in ASD is unclear. With accumulating ASD exome or whole genome sequencing being made public, leveraging previously reported DNVs is an effective strategy for clearly establishing the role of novel risk genes in ASD.

In this study, we implemented several approaches to tackle heterogeneity. First, we separated established ASD genes into network modules that likely represent more homogenous functions. The second was to leverage 5-HT as an endophenotype.

Genetic variants implicated in both hyperserotonemia and ASD are enriched in the subset of probands with hyperserotonemia so that we are equipped with increased power to detect ASD genes that function through regulating serotonin levels. Using this strategy in our data, we were able to identify novel candidate ASD genes not identified in previous large-scale studies that (we imagine) might poorly represent “hyperserotonemic ASD” risk factors. Although the significance is not striking in NGSEA, the non-overlapping LoF and Mis-D2 genes in the High-5HT group show enrichment in the same module (DAWN-1). In contrast, the genes harboring functional DNVs in the Normal-5HT group did not uncover *new* ASD genes in NGSEA, probably due to that fact that the majority of ASD patients have normal 5-HT, so that genes identified in previous large-scale studies are already more likely to represent the genes identified in the Normal-5HT probands studied here.

In this study we focused on DNVs and RAVs, two mechanisms that we hypothesized are involved in serotonin-related ASD genetic etiology. It is evident that signals due to DNVs are noticeably stronger than RAVs, presumably due in part to the larger effect sizes for DNVs and in part to the need to restrict analysis of RAVs to those from European subjects. For RAVs we observed significant enrichment with the TADA-1 list, which was derived by TADA’s joint modeling of both de novo and inherited variants in a previous study [11]. We analyzed homozygotes and CHs separately, and it is the CHs, not homozygotes, that showed significant enrichment. For example, all of the RAV genes overlapping with the TADA list are CHs, among which two genes (*ETFB* and *RELN*) are in the High-5HT group and two, a lysine methyltransferase and a calcium channel gene, are in the Normal-5HT group (*CACNA2D3* and *KMT2C*).

We note that the sample size of our study is small given the context of extensive genetic heterogeneity of ASD. For all association and enrichment analyses, we reported nominal *p* values without correcting for multiple testing. Varying degrees of dependency across tests makes adjustment for multiple comparisons a challenging problem, even when simulations are used to estimate significance empirically. Given the overall enrichment patterns in biologically relevant gene sets and pathways, our analyses provide promising candidates for further validation in large-scale studies.

## Conclusions

Our study identified novel ASD genes and provided further support for previously reported genes harboring DNVs implicated in ASD. Leveraging 5-HT as an endophenotype, we identified novel candidate genes related to the TGF-β pathway and cell junction function as contributing to serotonin-related ASD risk. Our study demonstrated the value of 5HT as an effective endophenotype in identification of novel ASD genes, warranting the collection of 5-HT in probands for future ASD genetic studies.

## Additional files

Additional file 1: Table S1. List of DNVs identified in our data. **Table S2.** Mean and standard deviation used for normalization of WB5HT. **Table S3.** Serotonin pathway genes. **Table S4.** Summary of subject demographics and phenotype data. **Table S5.** GSEA of LoF DNVs in ASC, SSC and ACE. **Table S6.** Gene lists disrupted by different types of DNVs in the High-5HT and the Normal-5HT groups. **Table S7.** GSEA of all RAVs and Normal-5HT RAVs. (XLSX 47 kb)

Additional file 2: Figure S1. The distribution of normalized 5-HT in parents and probands. The two vertical lines at 5-HT = 1.0 and 1.75 are the cutoffs used to define the High group (5HT > 1.75) and the Normal-5HT group (5HT < 1.0). (PDF 19 kb)

Additional file 3: Figure S2. The result of PCA indicates the threshold to identify the individuals with European ancestry. Left panel: PC plot of CEU, CHB, YRI from the 1000 Genome Project. Right panel: PC plot of our data. (PDF 8 kb)

Additional file 4: Figure S3. Gene length distribution of functional/disease gene sets used in GSEA. Most of gene sets have higher median gene length than the set of all genes (background distribution). The red line indicates the median length of all genes in the genome. (PDF 80 kb)

Additional file 5: Figure S4. Burden of RAVs in Fathers, Mothers and Probands. (a) All RAVs. (b) Homozygous RAVs. (c) Compound heterozygous RAVs. (PNG 430 kb)

## Abbreviations

5-HT: 5-Hydroxytryptamine
ACE: Autism Center of Excellence
ASC: Autism Sequencing Consortium
ASD: Autism spectrum disorder
CDS: Coding sequence
CH: Compound heterozygote
CIDR: Center for Inherited Disease Research
CNS: Central nervous system
CNV: Copy number variant
CRF: Chromatin remodeling factors
DAWN: Detecting Association with Networks
DEs: Differentially expressed genes
DNV: De novo variant
Dscore: Deleteriousness score
ECG: Evolutionarily constrained gene
FMRP: Fragile X mental retardation protein
GSEA: Gene set enrichment analysis
GWAS: Genome-wide association studies
hcASD gene: High confidence ASD gene
High-5HT: Group with 5-HT >1.75
HME: Histone modification enzyme
Hpsd: Postsynapse-related genes from proteomic profiling of human neocortical biopsies
LoF: Loss of function
MAGI: Merging Affected Genes into Integrated networks
Mis: Missense
Mis-D2: Missense variants with Dscore ≤2
NGSEA: Network-based gene set enrichment analysis
Normal-5HT: Group with 5-HT <1
PDN: Propagation-based Distance in Networks
PSD: Postsynaptic density
RAV: Recessively acting variant
RBFOX1: RNA-binding protein, fox-1 homolog 1
SNV: Single nucleotide variant
SSC: Simons Simplex Collection
WES: Whole exome sequencing
