# Supplementary figures and images for: Leveraging blood serotonin as an endophenotype to identify de novo and rare variants involved in autism
Source: Mol Autism. 2017 Mar 21;8:14. doi: 10.1186/s13229-017-0130-3 (PMC5361831; doi:10.1186/s13229-017-0130-3)

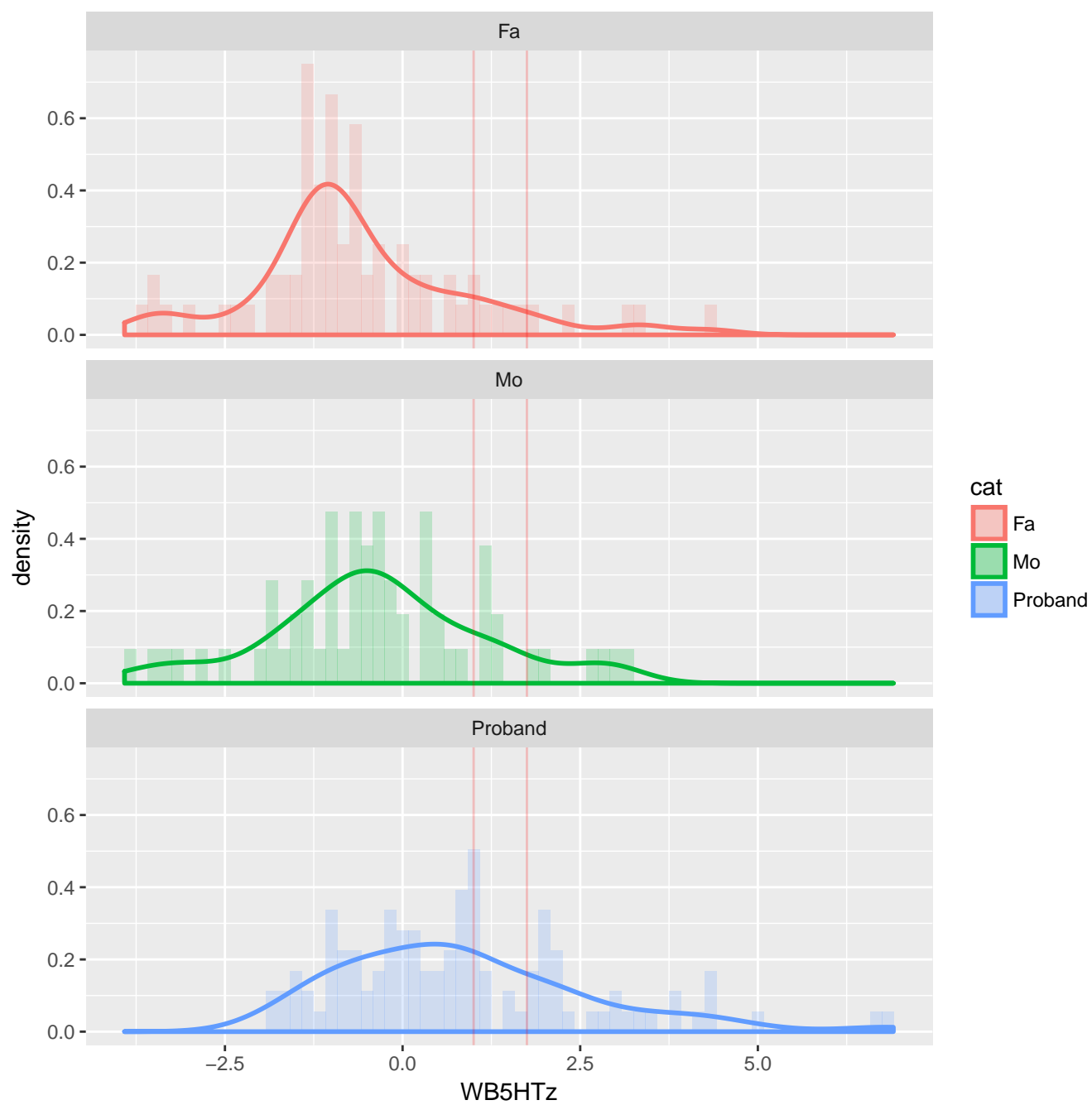

Supplement: Supplementary file 2 — The distribution of normalized 5-HT in parents and probands. The two vertical lines at 5-HT = 1.0 and 1.75 are the cutoffs used to define the High group (5HT > 1.75) and the Normal-5HT group (5HT < 1.0). (PDF 19 kb) [file 13229_2017_130_MOESM2_ESM.pdf]

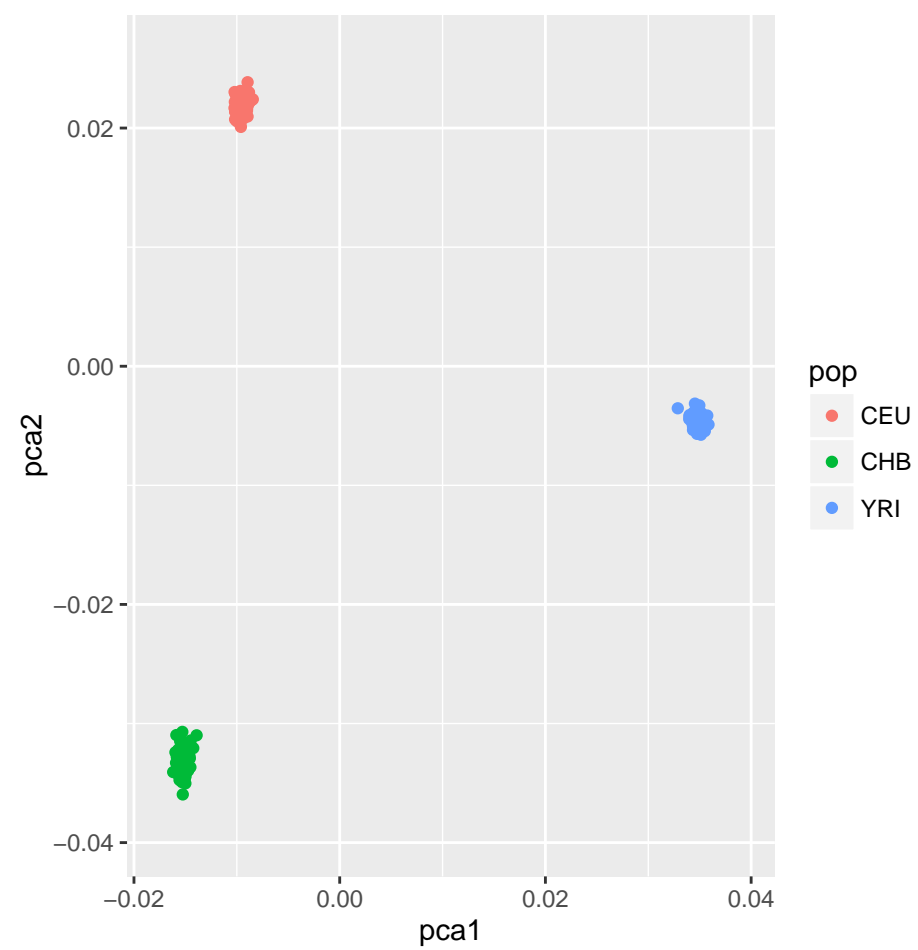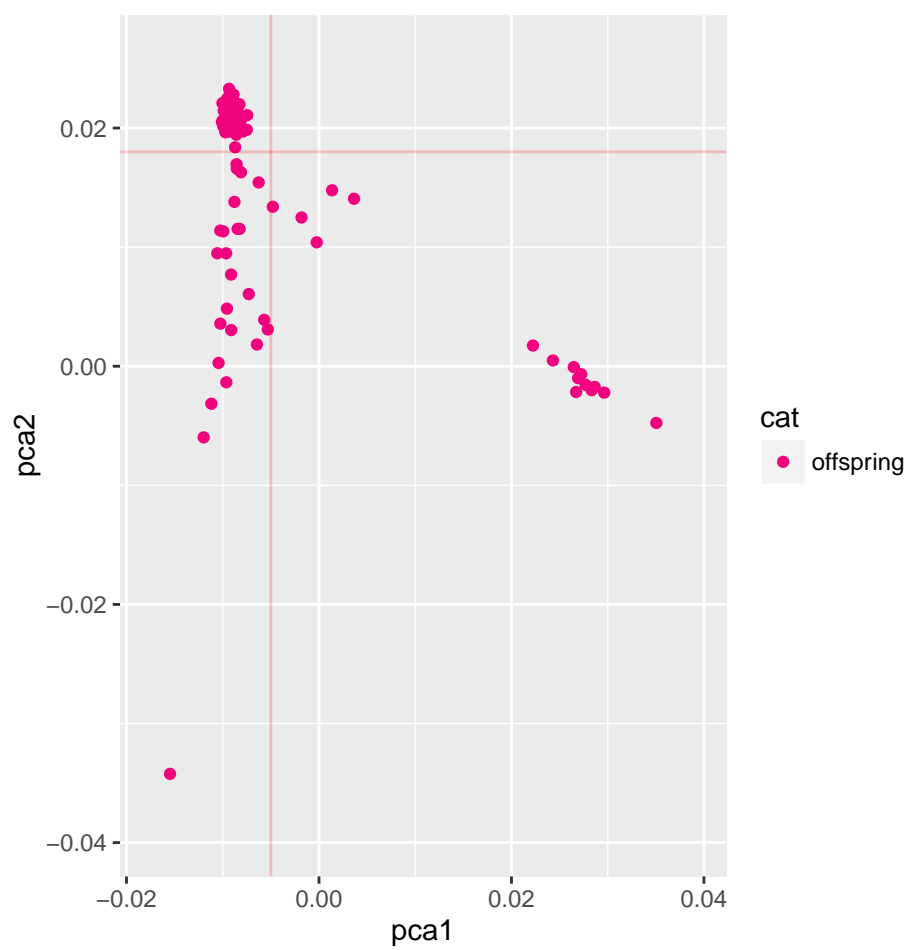

Supplement: Supplementary file 3 — The result of PCA indicates the threshold to identify the individuals with European ancestry. Left panel: PC plot of CEU, CHB, YRI from the 1000 Genome Project. Right panel: PC plot of our data. (PDF 8 kb) [file 13229_2017_130_MOESM3_ESM.pdf]

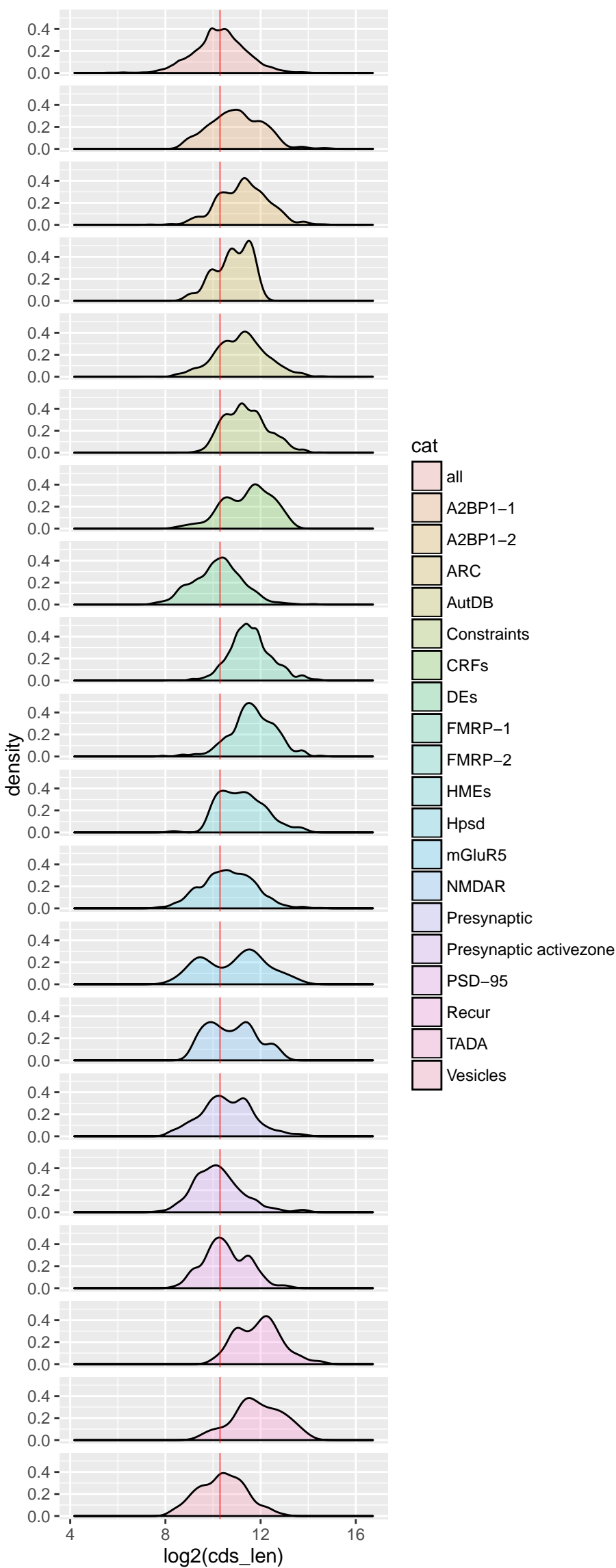

Supplement: Supplementary file 4 — Gene length distribution of functional/disease gene sets used in GSEA. Most of gene sets have higher median gene length than the set of all genes (background distribution). The red line indicates the median length of all genes in the genome. (PDF 80 kb) [file 13229_2017_130_MOESM4_ESM.pdf]

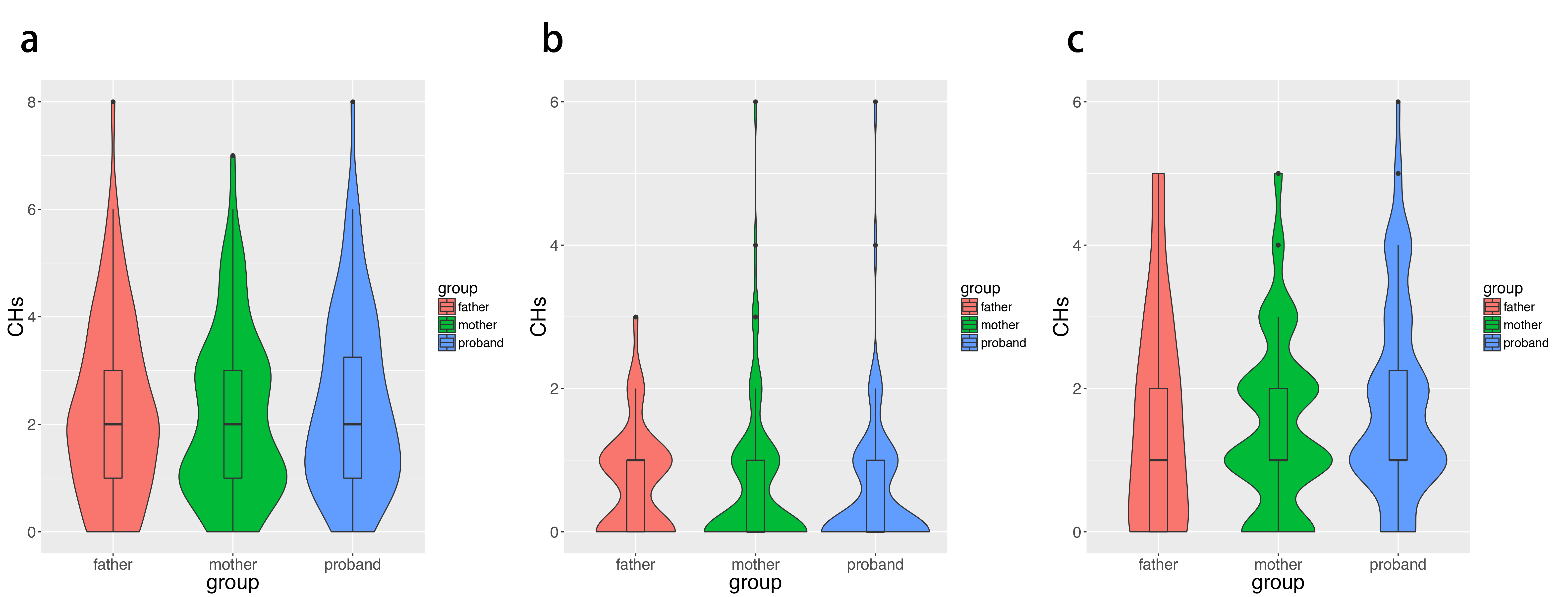

Supplement: Supplementary file 5 — Burden of RAVs in Fathers, Mothers and Probands. (a) All RAVs. (b) Homozygous RAVs. (c) Compound heterozygous RAVs. (PNG 430 kb) [file 13229_2017_130_MOESM5_ESM.png]
